# Polar Lipids of *Burkholderia pseudomallei* Induce Different Host Immune Responses

**DOI:** 10.1371/journal.pone.0080368

**Published:** 2013-11-18

**Authors:** Mercedes Gonzalez-Juarrero, Naoko Mima, Lily A. Trunck, Herbert P. Schweizer, Richard A. Bowen, Kyle Dascher, Waithaka Mwangi, Torsten M. Eckstein

**Affiliations:** 1 Department of Microbiology Immunology and Pathology, Colorado State University, Fort Collins, Colorado, United State of America; 2 Department of Biomedical Sciences, Colorado State University, Fort Collins, Colorado, United States of America; 3 Department of Veterinary Pathobiology, Texas A&M University, College Station, Texas, United States of America; The Scripps Research Institute and Sorrento Therapeutics, Inc., United States of America

## Abstract

Melioidosis is a disease in tropical and subtropical regions of the world that is caused by *Burkholderia pseudomallei*. In endemic regions the disease occurs primarily in humans and goats. In the present study, we used the goat as a model to dissect the polar lipids of *B. pseudomallei* to identify lipid molecules that could be used for adjuvants/vaccines or as diagnostic tools. We showed that the lipidome of *B. pseudomallei* and its fractions contain several polar lipids with the capacity to elicit different immune responses in goats, namely rhamnolipids and ornithine lipids which induced IFN-γ, whereas phospholipids and an undefined polar lipid induced strong IL-10 secretion in CD4^+^ T cells. Autologous T cells co-cultured with caprine dendritic cells (cDCs) and polar lipids of *B. pseudomallei* proliferated and up-regulated the expression of CD25 (IL-2 receptor) molecules. Furthermore, we demonstrated that polar lipids were able to up-regulate CD1w2 antigen expression in cDCs derived from peripheral blood monocytes. Interestingly, the same polar lipids had only little effect on the expression of MHC class II DR antigens in the same caprine dendritic cells. Finally, antibody blocking of the CD1w2 molecules on cDCs resulted in decreased expression for IFN-γ by CD4^+^ T cells. Altogether, these results showed that polar lipids of *B. pseudomallei* are recognized by the caprine immune system and that their recognition is primarily mediated by the CD1 antigen cluster.

## Introduction

Interest in the pathogenesis of infection with *Burkholderia pseudomallei*, the causative agent of melioidosis, and the related pathogen *B. mallei* which causes glanders disease, has substantially increased following their classification in the United States as category B priority and more recently Tier 1 pathogens with biothreat potential. While melioidosis has traditionally been recognized as an endemic disease in Southeast Asia and Northern Australia, it is now increasingly diagnosed in other tropical and subtropical regions of the world, including South and Central America, Africa and and the Indian subcontinent [1], . These findings are consistent with the notion that melioidosis is greatly under- or misdiagnosed and probably endemic throughout most of the subtropical and tropical regions of the world. Melioidosis is refractory to antibiotic therapy and requires aggressive and lengthy acute and eradication phase treatments [5]. In addition, recurrent infections with the same or different strains of *B. pseudomallei* are common after antibiotic treatment [6]. Furthermore, at present there are no licensed vaccines to prevent this infection. It is believed that lack of appropriate tools for diagnosis and lack of adequate treatment for chronic and/or recurrent melioidosis are responsible for the current epidemiological scenario of melioidosis. Thus, new strategies for diagnostics, therapeutics, and vaccines are needed.

Development of prophylactics for melioidosis requires definition of correlates of immune protection against *B. pseudomallei* infection. In the early stages of infection, NK and T cells are important but not essential [7], [8], [9], [10], [11], [12]. On the other hand IFN-γ, IL-12, and IL-18 cytokine expression are required, and it appears that multiple cell types release these cytokines early post-infection [8], [13], [14]. However, during the late stages of infection, antigen-specific T cells, mainly CD4^+^ T cells producing IFN-γ are essential for protection [8], [14], [15]. Some of these IFN-γ^+^CD4^+^ T cells have demonstrated the capacity to respond to specific *B. pseudomallei* proteins in mice and in humans [8], [9], [16], [17], [18], [19]. It has been suggested that the main function of IFN-γ is activation of macrophages, however the exact mechanism(s) involved in the production of IFN-γ during the infection or why there is incomplete protection against this pathogen are still unknown. In addition, it is unknown, which mechanisms counteract the protective antimicrobial effect generated by this cytokine. Finally, there is still a lack of knowledge on the potential role of immunosuppressive cytokine(s) and regulatory cells generated during *B. pseudomallei* infection, and these gaps have not been addressed in recent research efforts.

Bacterial cell envelopes are known to contain a wide array of lipids and the majority of them – phospholipids – are integral components of the cytosolic membrane. The amount and the variety of lipids are different and specific for each bacterial genus and/or species. While it is well known for mycobacteria, corynebacteria, and *Nocardia* spp. that their cell envelopes contain up to 40% of lipids, other bacteria like *Francisella* spp. or *Mycoplasma* spp. contain only low amounts and few distinct lipids. For a long time it was assumed that *B. pseudomallei* does not contain large amounts of lipids and that most of them are phospholipids and rhamnolipids, which were mostly viewed as exopolysaccharides but not lipids. However, recent studies revealed that the cell envelope of *B. pseudomallei* does not contain a wide array of lipids and carbohydrates. Several of these molecules are noted for immunological activities. Phung et al. (1995) identified a highly polar glycolipid, later identified as rhamnolipid that could be used as a diagnostic tool using sera from humans with melioidosis [20]. Four surface polysaccharides are proposed for *B. pseudomallei* according to genomic analyses and two of them were characterized in more detail: capsular polysaccharide (or type I O-PS) and type II O-PS [21], [22], [23]. The type II O-antigenic polysaccharide moiety of *B. pseudomallei* lipopolysaccharide is required for serum resistance and virulence [21], [22].

Bacterial lipids can be divided into non-polar lipids, phospholipids, and polar lipids. While knowledge about the function of non-polar lipids is limited, more is known about phospholipids because they are membrane constituents. Polar lipids on the other hand are increasingly being recognized as playing roles in host-pathogen interaction and as stimulators of humoral and cellular immune responses. In other bacterial infections, it has been described that lipids are presented by dendritic cells bearing the CD1 molecule and are recognized as specific antigens by CD1-restricted T cells [24], [25]. We hypothesized that characterization of the contingent of lipids of *B. pseudomallei* and their abundance would contribute to the understanding of the pathogenic mechanisms of this bacterium and the host's response to infection. Thus, in this study we demonstrated that polar lipids of *B. pseudomallei* presented by caprine DCs can elicit immune T cell responses and that CD1w2 (CD1b antigens in humans) antigens participate in this process.

## Materials and Methods

### Chemical Reagents

All chemical reagents were of the highest grade available and obtained from Sigma-Aldrich, LLC (St. Louis, MO) unless otherwise specified.

### Lipid isolation and antigen preparation

*B. pseudomallei* strain K96243 [26] was grown for 16 h in Middlebrook 7H9 medium supplemented with glucose at a final concentration of 0.2%. The wet cell pellet was treated with chloroform/methanol (1∶1) at a ratio of 10∶1 (vol/vol) for seven days at 4°C. This unusual extraction time was necessary to ensure that the bacteria were dead and the mixture could be transferred out of the BSL-3 laboratory. After centrifugation the supernatant was used for the first extraction containing all cell envelope lipids (termed F1) and the remaining cell debris pellet was dried under nitrogen. A second extraction was performed on dried residue cell debris with chloroform/methanol (2∶1) and the supernatant from this extraction was used for the second extraction, which contained only polar lipids (termed F2). Each one of the extractions was purified by Folch-washing with 6 ml chloroform/methanol (2∶1) and 1 ml of water. The organic phase was transferred to a new pre-weight tube and dried down under a constant stream of nitrogen. The extracted and purified lipids were suspended in chloroform/methanol (2∶1) to a concentration of 10 mg/ml. Lipids were analyzed and separated by two dimensional thin layer chromatography (2D-TLC) in five different solvent systems on aluminum-backed silica 60 F_254_ gel plates (EMD Chemicals, Gibbstown, NJ) (**Table 1**). Lipids were visualized by spraying with 10% CuSO_4_ in 8% H_3_PO_4_ followed by heating or by exposure to UV light. Lipids were further analyzed by differential spraying of the plates to detect carbohydrates (α-naphthol solution), primary, secondary, and tertiary amino groups (using ninhydrin spray solution), and negatively charged groups (primarily phosphates) (using Dittmer-Lester spray solution).

**Table 1:** Solvent systems used for two-dimensional thin layer chromatography.

Polarity	Name	1^st^ Dimension	2^nd^ Dimension
Polar	B	Chloroform/methanol/water (60∶30∶6)	Chloroform/acetic acid/methanol/water (40∶25∶3∶6)
**↓**	A	Chloroform/methanol/water (100∶14∶0.8)	Chloroform/acetone/methanol/water (50∶60∶2.5∶3)
	E	Chloroform/methanol (96∶4)	Toluene/acetone (80∶20)
	C	Petroleum ether/acetone (92∶8)	Toluene/acetone (95∶1)
Apolar	D	Petroleum ether/ethyl acetate (98∶2)	Petroleum ether/acetone (98∶2)

Material from the second extraction contained only polar lipids and was further fractionated on a silica 60 gel column with increasing concentrations of methanol in chloroform: 67% for fraction V (termed F4), 75% for fraction VI (termed F5), 80% for fraction VII (termed F6), and 90% for fraction VIII (termed F7). Lipid fractions were purified by Folch wash, which also removes endotoxin. For the preparation of each tested lipid fraction endotoxin-free water and solutions were used. Glassware for lipid preparation was endotoxin-free which was archived by heating the glassware at 450°C for at least 8 h. All chemicals used were obtained as endotoxin-free from Sigma-Aldrich, LLC (St. Louis, MO). One hundred micrograms of each of the *B. pseudomallei* polar lipid fractions and lipid extracts were first dissolved in 10 µl iso-propanol followed by addition of 90 µl Roswell Park Memorial Institutes 1640 medium (RPMI). This stock solution was further diluted with RPMI 1640 media to a final concentration of 0.4 µg/ml. These final solutions were used as antigens in caprine dendritic cells (cDCs) - T cell co-culture stimulation assays. Concanavalin A (ConA) (5 µg/ml) mitogen was used as the positive control, whereas complete RPMI 1640 medium (cRPMI) (RPMI 1640 medium containing 10% fetal bovine serum, 2 mM L-glutamine, 100 mM HEPES, penicillin [100 IU/ml], streptomycin [100 µg/ml] and gentamicin [50 µg/ml], and 5×10^–5^ M 2-mercaptoethanol) served as the negative control.

### MALDI-TOF of lipid fractions and spots

Matrix-assisted laser desorption ionization-time of flight (MALDI-TOF) was performed in the Proteomics and Metabolomics Facility, Colorado State University, with an Ultraflex MALDI/TOF/TOF (Bruker Daltonics, Billerica, MA) with 2,5 dihydrobenzoic acid and NaI as the matrix.

### Animal Experiments

For proof-of-concept, two female adult Nubian breed goats, 12 months (#19) and 36 months (#29) of age, were obtained from a disease-free herd and used for these studies. Both animals remained healthy throughout the study. Animals were bled every two weeks for the duration of the study (18 months). This study was carried out in strict accordance with the recommendations in the Guide for the Care and Use of Laboratory Animals of the National Institutes of Health. The protocol was approved by the Institutional Animal Care and Use Committee (IACUC) of Colorado State University (Approval # 09-1507A).

### Blood collection

Jugular venous blood (15–20 ml) was collected from goats into EDTA tubes as needed. White blood cells were prepared by lysis of red blood cells with Gey's solution. Whole blood was incubated with Gey's solution for 5 minute followed by PBS. The mixture was then centrifuged and the cell pellet washed three times with PBS. Cells were re-suspended to a final concentration of 2×10^6^ cells/ml in cRPMI medium.

### Preparation of dendritic cells

Blood-derived caprine dendritic cells (cDCs) were prepared by culture differentiation of monocytes obtained by plastic adhesion of white blood cells. Briefly, the white blood cells were incubated for at least 2 h in cRPMI medium at 37°C, 5% CO_2_. Thereafter, non-adherent cells were removed and adherent monocytes were rinsed with sterile PBS. Adherent monocytes were incubated for 7 d at 37°C, 5% CO_2_ in cRPMI medium containing 2 ng/ml of a cocktail of cytokines containing a chimeric recombinant bovine Flt3L/GM-CSF and bovine IL-4 [27]. The recombinant cytokines used in this protocol were expressed as FLAG-tagged proteins in HEK 293 Free-Style cells (Invitrogen) and affinity-purified using anti-FLAG M2-agarose beads (Sigma-Aldrich). Live cDCs were visualized and images were captured using a LSM 510 Zeiss confocal microscope. The surface antigen phenotype of the cDCs was determined by flow cytometric analysis using monoclonal antibodies (mAbs) specific for anti-bovine CD1w2 (CC20; AbD Serotec), mouse anti-ovine MHC class II DR (AbD Serotec) and anti-human CD14 (M5E2; BioLegend). Caprine dendritic cells were stimulated for 16 h with each one of the polar lipid fractions described above. For this purpose, the cDCs (1×10^5^ cells/well) were incubated with each one of the polar lipid fractions at 0.4 mg/ml in a total volume of 100 µl complete RPMI. In those experiments aiming to block the CD1w2 antigen, cDCs pulsed with each of the lipid fractions were pre-incubated for 1 h with purified anti-CD1w2 antibodies prior to adding T cells.

### T cell isolation

T cells present in the white blood cells were isolated using nylon wool columns as reported earlier [28]. Briefly, white blood cells (5–6×10^7^ cells/10 ml of cRPMI) were loaded onto sterile nylon wool columns and incubated for 30 min at 37°C. Then the columns were placed in a support and T cells (non-adhered to nylon wool) were recovered by eluting the columns using cRPMI medium and the cells were concentrated by centrifugation. Thereafter, cells were stained with fluorescence labeled mAbs against CD4 and CD8 T cell markers (AbD Serotec). This procedure resulted in approximately 85% enrichment of the T cells from the original blood white cell suspension. Isolated T cells were co-cultured with antigen-pulsed cDCs [at a ratio of 5∶1] for 72 h or 7 d depending on the assays. After 72 h some co-cultures received 2 ng/ml of bovine IL-2 (Kingfisher, Biotech Inc.) and they were incubated for 5 d after which intracellular cell cytokine assays were performed as explained below.

### T cell stimulation studies

In some experiments, T cells were labeled with carboxyfluorescein diacetate succinidyl ester (CFSE) dye [CellTRAce™CFSE dye, Invitrogen] following the manufacturer's recommendations. CFSE-labeled T cells were used to determine the antigen specific proliferation capacity of CD4^+^ T cells in the presence of cDCs and each polar lipid fraction extracted from *B. pseudomallei*. After 72 h of culture, the cells were harvested and stained with fluorescence labeled anti-ovine CD4 mAb (clone 44.38; AbD Serotec). Thereafter, cells were evaluated by flow cytometry and CD4^+^ T cell proliferation was determined by measuring the loss in the intensity of CFSE fluorescence. ConA (5 µg/ml) treated cells served as the positive control, whereas CFSE-labeled T cells cultured in medium alone served as the negative control. In another assay, unlabeled T cells and antigens were added to cDCs and 72 h later cells were harvested and subjected to cell surface staining for T cell activation markers using fluorescence labeled anti-ovine CD4 mAb and anti-bovine CD25 mAb (clone 9.14) (AbD Serotec).

The profile of cytokines expressed by T cells after 7 d of culture in the presence of autologous cDCs and each of the polar lipid fractions extracted from *B. pseudomallei* was determined by intracellular staining using fluorescence anti-IFN-γ or IL-10 mAbs (AbD Serotec). For this purpose, prior to the staining procedure, each culture was incubated with leukocyte activating cocktail containing Golgi Plug (BD Biosciences) for 6 h as per manufacturer's instructions and then the cells were stained with the fluorescence labeled anti-ovine CD4 mAb. Thereafter, the cells were incubated with BD Cytofix/Cytoperm reagents (BD Bioscience) and stained with antibodies against bovine IFN-γ (clone CC302), mouse IL-10 (clone JES5-2A5), or mouse and rat negative controls IgG1 (AbD Serotec) to determine the cells producing these cytokines. The stained cells were tested by Flow Cytometry Cyan (Beckman Coulter) and the data acquired was analyzed using the FlowJo software (TreeStar, Ashland, OR). Gate strategy included selection of cell populations according to their side/forward scatter profile. Thereafter, CD4^+^ T cells with positive cytokine staining for each gate was compared to their corresponding isotype-matched control and the percentage of positive cells and intensity of fluorescence was recorded as percentage of positive cells and mean fluorescence channel (MFC), respectively. The significance of the difference (presented as the percentage of positive cells) between co-cultures of *B. pseudomallei* lipid-pulsed cDCs with T cells and the negative control co-cultures was determined by statistical analysis of the Overton subtraction of percentage of positive cells using the FlowJo software.

## Results

### Total lipids of *B. pseudomallei*

**Figure 1:**
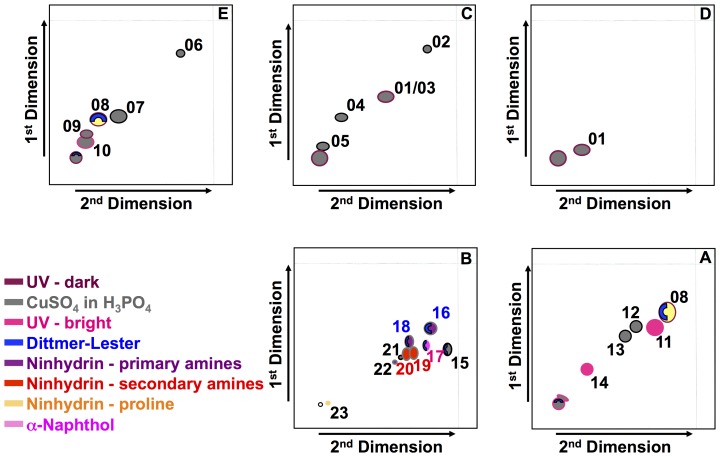
Composite sketch of total lipids of *B. pseudomallei*. Lipids shown were found within the first extract in five different solvent systems from very polar to highly non-polar (B, A, E, C, D). Colors of various lipid spots represent different staining and detection methods: Maroon Red: Spots detected as dark spot under UV light; Strawberry Red: Spots detected as a bright spot under UV light; Steel gray: Spots detected by spraying with CuSO_4_ in H_3_PO_4_; Blue: Spots detected by spraying with Dittmer-Lester reagent; Plum purple: Spots detected by spraying with ninhydrin as molecules containing primary amino groups; Red: Spots detected by spraying with ninhydrin as molecules containing secondary amino groups; Orange: Spots detected by spraying with ninhydrin as molecules containing tertiary amino groups/proline; Magenta: Spots detected by spraying with α-naphthol.

Total lipids of *B. pseudomallei* (termed F1) were obtained through the first extraction of cell lipids. There were at least 23 different lipid spots identified by two-dimensional thin layer chromatography within the five different solvent systems (**Figure 1**). While most of the non-polar lipids appeared only under UV light all other lipid spots could be visualized by the general stain with CuSO_4_ in H_3_PO_4_.

### Polar lipids of *B. pseudomallei*

**Figure 2:**
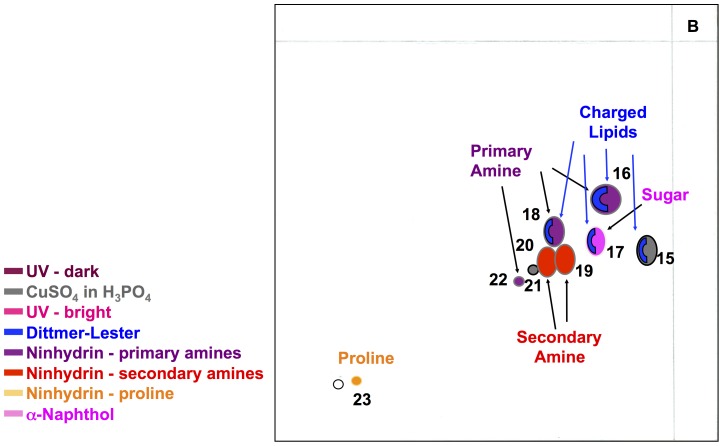
Composite sketch of polar lipids of *B. pseudomallei*. Lipids are shown as found within the second extract in the highly polar solvent system B. Colors of various lipid spots represent different staining and detection methods: Maroon Red: Spots detected as dark spot under UV light; Strawberry Red: Spots detected as a bright spot under UV light; Steel gray: Spots detected by spraying with CuSO_4_ in H_3_PO_4_; Blue: Spots detected by spraying with Dittmer-Lester; Plum purple: Spots detected by spraying with ninhydrin as molecules containing primary amino groups; Red: Spots detected by spraying with ninhydrin as molecules containing secondary amino groups; Orange: Spots detected by spraying with ninhydrin as molecules containing tertiary amino groups/proline; Magenta: Spots detected by spraying with α-naphthol.

Polar lipids (termed F2) were obtained through the second extraction from the *B. pseudomallei* cell envelope. Two-dimensional thin layer chromatography (2D-TLC) identified nine lipid spots when using the most polar solvent system B (**Figure 2**). Differential staining of 2D-TLCs demonstrated the presence of charged lipids (lipid spots 15 to 18), sugar containing lipids (lipid spot 17), and lipids with primary amino groups (lipid spot 16, 18, and 22), secondary amino groups (lipid spots 19 and 20), or tertiary amino groups including proline (lipid spot 23). Further fractionation of the second extract (polar lipids) resulted in four significant fractions with either single lipid spots or only few lipids spots (**Figure 1C**). Fraction F4 contains only lipid 16 while fraction F7 contains only lipid 19. In contrast to these two fractions, fractions F5 and F6 were a mix of a few lipids with but did not contain lipid spots 16 and 19. In particular, fraction F5 contained lipid spots 16, 17, and 18, while fraction F6 contained lipid spots 16, 18, 19. Lipid spot 17 was the only lipid spot that stained positive with α-naphthol indicating that it contains the glycolipids known as rhamnolipids, the only known glycolipids of *B. pseudomallei*. Lipid spot 16 stained positive for primary amino groups and positive for charged lipids. Lipid spot 18 contains charged lipids and the molecular ions suggest that it contains the well-known inner membrane phospholipid phosphatidylethanolamine (PE). Lipid spot 19 could not be further characterized. However, we did determine that lipid spot 19 contains a secondary amino group and it is neither a charged lipid nor a glycolipid.

### MALDI-TOF of lipid fractions and lipid spots

**Figure 3:**
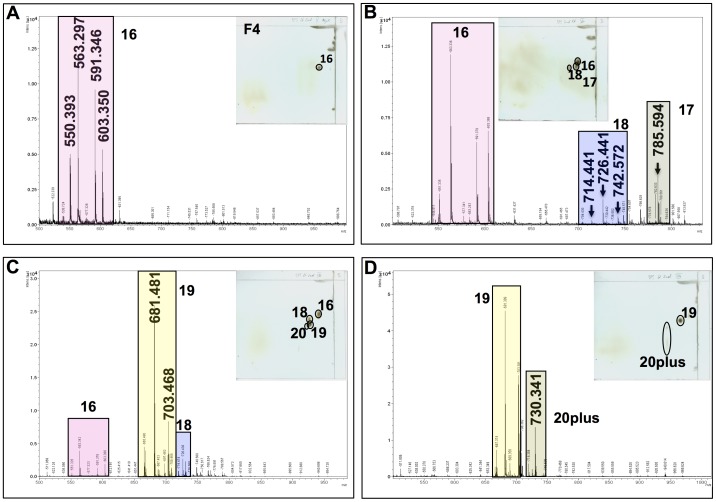
Mass spectrometry of the four major fractions of polar lipids. Lipids derived from the second lipid extract of *B. pseudomallei*. Each mass spectrometric trace has the lipid composition of its fraction indicated by a 2D-TLC plate in the upper right corner. Lipid spot numbers correspond to those used in Figures 1 and 2. Major ions of each spot are highlighted in colored boxed. Molecular ions are in bold and enlarged in panels they first appeared. Panel A represents lipid fraction V (F4); panel B represents lipid fraction VI (F5); panel C represents lipid fraction VII (F6); and panel D represents lipid fraction VIII (F7).

**Figure 4:**
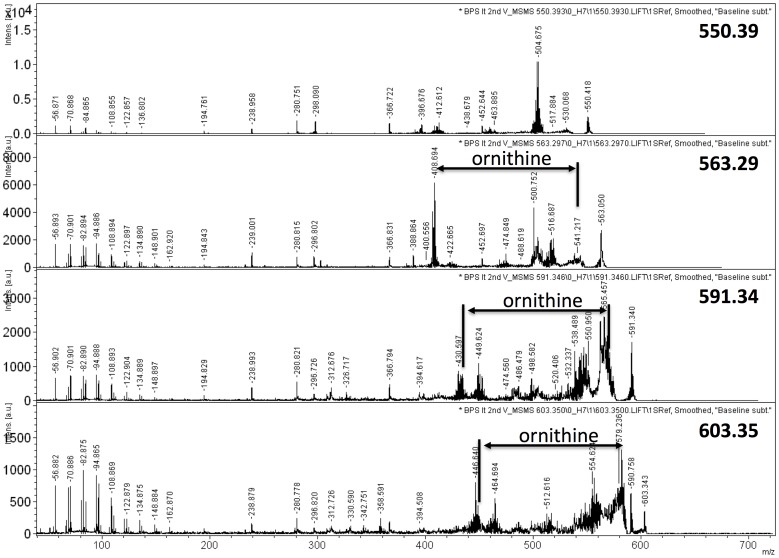
Mass spectrometry analysis by MALDI-TOF-TOF of the four major molecular ions of lipid spot #16. Arrows highlight the difference between two major ions representing the loss of ornithine.

Mass spectrometry (MS) and MS/MS were performed by MALDI-TOF using sodium 2,5 dihydrobenzoate resulting in primarily sodium-based positive ions. MALDI-TOF was performed for lipid spots 16 to 19 and polar lipid fractions V to VIII (Figures 2, 3, 4). MALDI-TOF MS data were combined with MS/MS data of key ions within each polar lipid fraction.

**Figure 3A** shows the mass spectrometry of primarily lipid spot 16, which is dominated by the ions of 563.30, 591.34, and two minor ion of 550.39 and 603.35 u. MS/MS analyses demonstrated clearly the presence of ornithine lipid within the ions of 563.30 u, 591.34 u, and 603.35 u (M+Na^+^) (**Figure 4**). Fragmentation of this ion resulted in the loss of ornithine (132.5 u) and yielded fragmentation ions of 408.69 u, 436.74 u, and 448.75 u, respectively. Thus, the predicted preliminary structure most likely consists of one C_12_ and one C_14_ fatty acyl group with one of them being 3-hydroxylated, while the other moiety is bound to the ornithine. The molecular ion of 591.34 would thus represent an ornithine lipid with one C_14_ and one 3-OH C_14_ fatty acyl group, while the molecular ion of 603.35 contains a mono-unsaturated fatty acid and saturated fatty acid. One of these lipids is hydroxylated. The lengths of the fatty acyl chains should be C_14_ and C_15_.

**Figure 5:**
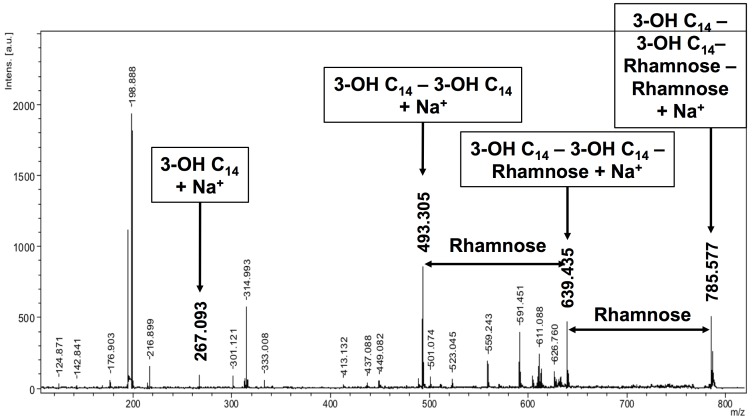
Mass spectrometry analysis by MALDI-TOF-TOF of the key molecular ions of lipid spot #17. Arrows highlight the differences between two major ions representing the loss of rhamnose. Molecular ions representing fragmentation ions are labeled.

**Figure 3B** shows the MALDI-TOF MS data of lipid spots 16, 17, and 18. Although already demonstrated by differential staining for carbohydrates, MALDI-TOF MS/MS for lipid spot 17 (molecular ion of 785.59 u) demonstrated the presence of a C_14_/C_14_ di-rhamnolipid (**Figure 5**). Our data confirm the previously determined presence and chemical structures of rhamnolipids of *B. pseudomallei* [22], [23], [29], [30]. The key molecular ion of spot 18 was the ion of 726.44 u. Differential staining of lipid spot 18 pointed already in the direction of putative phospholipids within spot 18 and indeed ions for C_16_/C_18_ PE (742.57 u), C_16_/C_16_ PE (714.47 u), and C_16_/C_17∶1_ PE (726.44 u) could be identified.

**Figure 3C and D** show the presence of lipid spot 19 and to some extent spot 20 in fractions VII and VIII. Differential staining of both lipid spots did not reveal any specific moieties within the lipids of these spots except that the molecules in these spots contain secondary amino groups.

### Characterization of caprine blood derived dendritic cells

**Figure 6:**
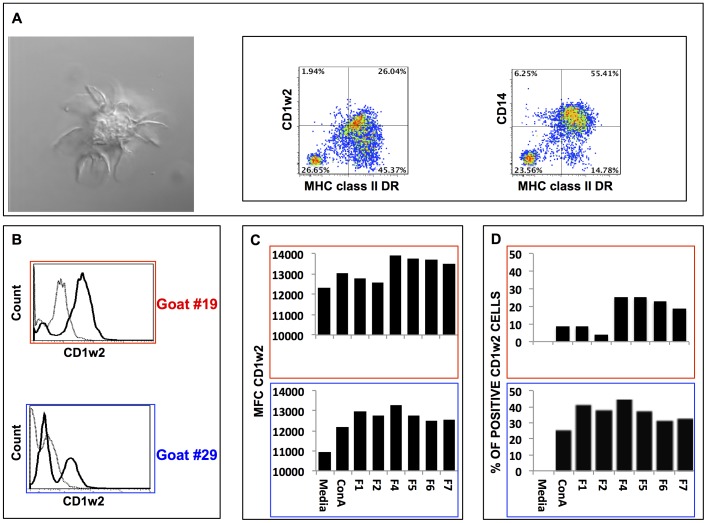
Characterization of caprine peripheral blood monocyte-derived dendritic cells. Panel A shows a photograph of a classic morphology of a caprine dendritic cell. The same cultures were examined for cell surface expression of myeloid dendritic cell markers: CD14, MHC class II DR, and CD1w2 antigens. In panel B are representative histograms for cell surface expression of CD1w2 antigen (dark line) or isotype matched control (dotted line) in caprine dendritic cells from goat #19 (top and framed in red) and goat #29 (bottom and framed in blue) when cultured in media alone. Panels C and D correspond to cell surface expression of CD1w2 antigen in caprine dendritic cells when cultured for 16 hours in culture media alone or culture media with ConA, first extraction of total cell lipids of *B. pseudomallei* (F1), second lipid extract (F2), or lipid fraction of the second lipid extract (F4 to F7). Panel C represents the mean Fluorescence Channel (MFC) for CD1w2 expression in each culture. Panel D represents the percentage of positive cells for CD1w2 when compared to media alone.

It is known that presentation of lipids to T cells is mediated by dendritic cells (DC) expressing the CD1 cluster of antigens [26]. Among those, the CD1b and CD1c have demonstrated capacity to present lipids to T cells and play a role in protection against mycobacteria [25]. We prepared cultures of monocyte-derived caprine DC (cDCs) from goat peripheral blood. Goat blood adherent monocytes were cultured in the presence of recombinant bovine GM-CSF-FLt3L and recombinant bovine IL-4 for 7 days. The cells in these cultures demonstrated classic morphology of dendritic cells when examined by confocal microscopy (**Figure 6A**). Cells from these cultures were also characterized by flow cytometric analysis for expression of the monocyte marker CD14 and the well-described DC subset markers CD1w2 (also known as CD1b) and MHC class II DR molecules. To detect these molecules on the surface of cDCs we used fluorescent labeled anti-bovine CD1w2 and anti-ovine MHC class II DR mAbs. These experiments demonstrated that cells in these cultures were positive for CD14, CD1w2 and MHC class II DR antigens (**Figure 6A**). Thus, we concluded that, as expected, goat adherent monocytes in the presence of a cocktail of bovine Flt3L/GM-CSF and bovine IL-4 differentiated into monocyte-derived DCs.

**Figure 7:**
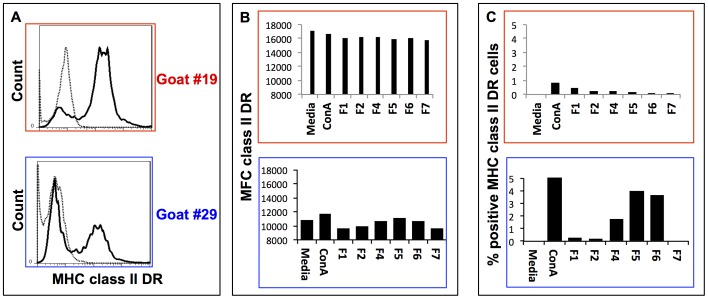
Surface expression of MHC class II DR antigens on caprine dendritic cells and effect of polar lipid fractions on surface expression for these molecules. Panel A shows representative histograms of surface expression of MHC class II DR molecules on cDCs cultured (dark line) or isotype matched control (dotted line) with culture media alone. Panels B and C correspond to cell surface expression of MHC class II DR molecule on cDCs when cultured for 16hr in culture media alone or culture media containing ConA or each of the polar lipid fractions (F1 to F7) extracted from *B. pseudomallei* as described in Figure 1. Panel C represents the Mean Fluorescence Channel (MFC) for MHC class II DR antigen expression in each cell culture. Panel D represents the % of positive cells for MHC class II DR antigen over the percentage of positive cells expressing MHC class II DR antigen on cDCs cultured in media alone.

Furthermore, we studied whether each of the polar lipid fractions extracted from *B. pseudomallei* had any effect on the surface expression of CD1w2 and MHC class II DR antigens on cDCs. For this purpose, cDCs were incubated for 16 h with each of the polar lipid fractions and the cell surface expressions of CD1w2 and MHC class II DR antigens were determined by flow cytometry. The surface expression for CD1w2 molecules on cDCs was up regulated by all polar lipid fractions when compared to similar cDCs in control cultures (**Figure 6B and C**). However, the expression of MHC class II DR antigens on cDCs recovered from the same cultures was down regulated or did not change when compared to similar cDCs cultured in medium alone (**Figure 7**). Interestingly, cDCs derived from goat #19 and cultured in the presence of polar lipids derived from fractions F4 – F7 had the highest increase in mean MFC and percentage of positive cells for CD1w2 antigens when compared to the expression of the same antigen in cells recovered from cultures containing F1 or F2 extracts, total and polar lipids, respectively (**Figure 6C**). The MFC and percentage of expression of CD1w2 and MHC class II DR varied between cultures of cDCs obtained from different animal subjects but followed similar trend of changes between animals.

### T cell responses to caprine dendritic cells pulsed with each of the *B. pseudomallei* lipid fractions

**Figure 8:**
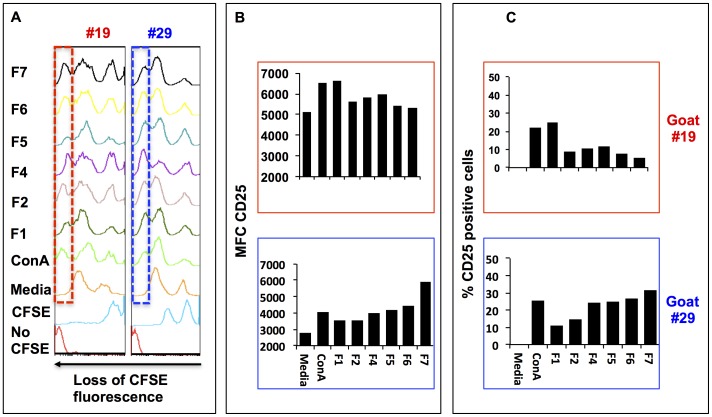
Autologous T cell activation by caprine dendritic cells. Autologous T cells were pulsed with media alone, ConA, first lipid extraction (F1), second lipid extraction (F2), and lipid fractions of F2 (F4 to F7). Panel A shows the proliferation of CD4 T cells when co-cultured with cDCs pulsed with *B. pseudomallei* polar lipid fractions (F1 to F7). The vertical dashed box within the histograms represents the threshold expression of CFSE below which cells were considered to have lost more fluorescence for the CFSE dye than CD4^+^ T cells harvested from the cultures containing media alone. Panel B represents the Mean Fluorescence Channel (MFC) for CD25 antigen expression in each cell culture. Panel C represents the percentage of positive cells for CD25 antigen over the percentage of positive cells expressing CD25 antigen on cDCs cultured in media alone obtained by Overton statistical analysis using the FlowJo software package. Upper and lower histograms or bar graphs correspond to animal #19 and #29, respectively.

The capacity of lipids to stimulate T cells is an important step towards activation of the acquired immune response. Priming of T cells requires up regulation of the CD25 molecules (IL-2 receptor), IL-2 expression and subsequent cell proliferation. Thus, using flow cytometry, we measured the capacity of T cells to proliferate and to up regulate expression of the CD25 molecules in response to *B. pseudomallei* polar lipids present in each of the lipid fractions. First, we measured the capacity of cDCs pulsed with each of the polar lipid fractions to stimulate proliferation of T cells. Proliferation in these cultures was determined by measuring the loss of fluorescence in the CFSE labeled CD4^+^ T cell population (**Figure 8A**). The results demonstrated that CD4^+^ T cells recovered after 72 h from co-cultures containing cDCs and CFSE-labeled T cells in the presence of each polar lipid fraction demonstrated higher proliferation than similar CD4^+^ T cells in control co-cultures. Similar co-cultures assays demonstrated that the percentage of CD4^+^ positive T cells expressing CD25 antigens increased between 10 and 20% in cells recovered from co-cultures containing *B. pseudomallei* polar lipid fractions than similar cells obtained from control co-cultures (**Figure 8B and C**). Again, changes in the percentage of positive cells and MFC varied between cell cultures derived from different animal subjects but similar trends of proliferation and CD25 expression were observed in cells derived from both goats.

### Cytokine profile of T cells recovered from co-cultures containing each one of the *B. pseudomallei* polar lipid fractions

**Figure 9:**
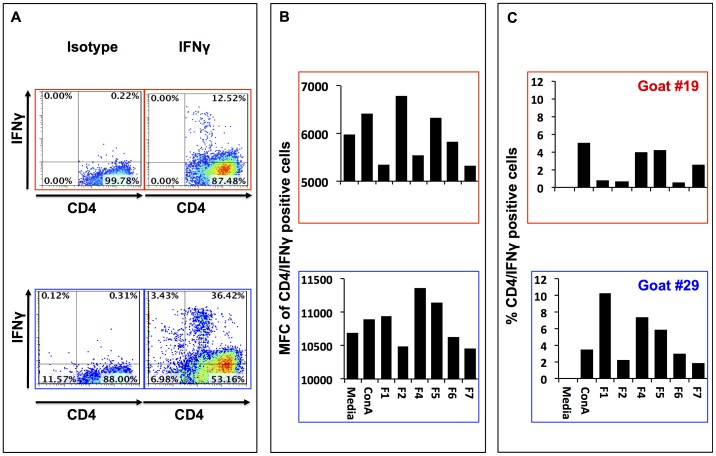
IFN-γ expression in CD4^+^ T cells. Panel A shows representative flow cytometry plots demonstrating positive staining for CD4 and IFN-γ staining in cells recovered from co cultures of autologous T cells and cDCs with fraction F4 extracted from *B. pseudomallei*. Panel B represents the Mean Fluorescence Channel (MFC) for CD4/IFN-γ positive cells in each cell culture. Panel C represents the % CD4/IFN-γ positive cells over the percentage of positive cells expressing CD4/IFN-γ cultured in media alone.

**Figure 10:**
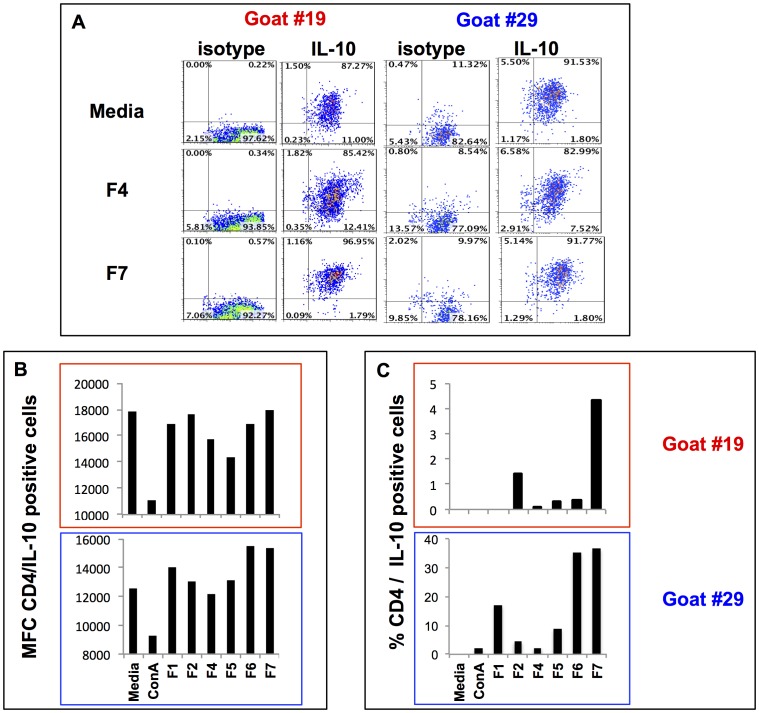
IL-10 expression in CD4^+^ T cells. Panel A shows the representative flow cytometry plots demonstrating positive staining for CD4 and IL-10 staining in cells recovered from co cultures of autologous T cells and cDCs with media alone, fraction F4 and F7 extracted from *B. pseudomallei*. Panel B represents the mean fluorescence channel (MFC) for CD4/IL-10 positive cells in each cell culture. Panel C represents the percentage CD4/IL-10 positive cells over the percentage of positive cells expressing CD4/IL-10 cultured in media alone.

The profile of cytokines expressed by T cells upon stimulation, in this case IFN-γ and IL-10, is likely to determine the outcome of the acquired immune responses towards a protective state of the host. For this reason we screened each of the *B. pseudomallei* polar lipid fractions for their capacity to stimulate IFN-γ and/or IL-10 expression by T cells recovered from these co-cultures. Cells recovered from each of the co-cultures were stained for co-expression of CD4 and IFN-γ (**Figure 9**) or IL-10 (**Figure 10**) cytokines. Flow cytometric analysis of T cells co-cultured for 7 d with cDCs pulsed with *B. pseudomallei* polar lipid fractions showed that CD4^+^ T cells expressed IFN-γ and IL-10 (**Figures 9** and **10**). Depending on the culture conditions, and when compared with their isotype-matched controls, 10–40% IFN-γ^+^CD4^+^ T cells were detected in each co-culture (**Figure 8A**). Comparison of IFN-γ expression by CD4^+^ T cells between the treatment and the controls revealed that only cells co-cultured in the presence of polar lipid contained in fraction F5 (in both goats #19 and #29), and polar lipids contained in fraction F4 and F2 (only in goat #29) had increased MFC (**Figure 9B**). Interestingly, cells in co-cultures stimulated with any of the lipids fractions contained higher percentage (between 1–7%) of IFN-γ^+^CD4^+^ cells than in control co-cultures. Furthermore, IFN-γ^+^CD4^+^ cells from co-cultures pulsed with polar lipids from fractions F4 and F5 had the highest percentage (4–7%) of positive cells among all the lipid fractions tested (**Figure 9C**). Cells from these co-cultures tested positive for IL-10 expression when compared to the control (**Figure 10A**). In this case, polar lipids from fraction F7 followed by F6 have the highest MFC and higher percentage of CD4/IL-10 positive cells (20–35%) when compared to cells harvested from control cultures (**Figure 10B**).

### CD1w2 contribution to IFN-γ expression by CD4^+^ T cells

**Figure 11:**
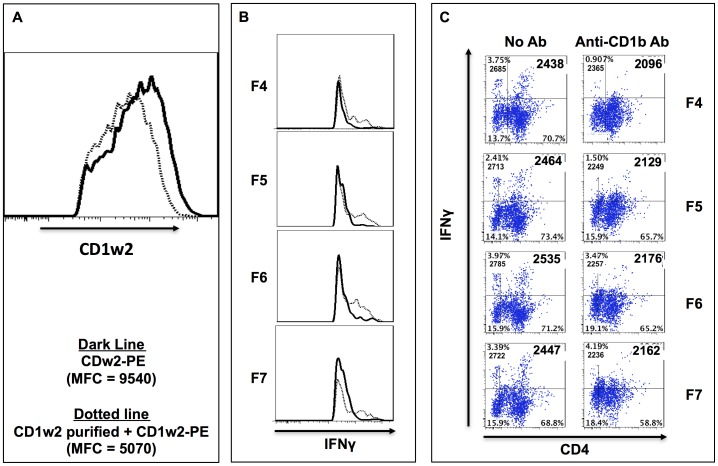
CD1w2 blocking effects on IFN-γ expression by CD4+ T cells when stimulated with polar lipids. Panel A show the histogram representing the MFC for CD1w2-PE antibody bound to cDC cultures incubated with fluorescence -CD1w2 antibody-PE (thick line, MFC = 9540) or with purified CD1w2 antibody plus CD1w2 –PE antibody [thin-dotted line, MFC 5070]. Panels B and C show representative histograms (B) and dot plots (C) of CD4 T cells expressing IFN-γ recovered from cultures after seven days incubation where the cDCs primed with each of the polar lipids when incubated with CD1w2 antibody (right column of dot plots) or without (left column of dot plots). Numbers inside the quadrants indicated the percentage (upper number) and the MFC (lower number) for IFN-γ expression in the CD4+ (right quadrants) and CD4- negative cells (left quadrants).

Contribution of the CD1w2 expressed on the surface of cDCs during the presentation of polar lipids contained in fractions F4 – F7 to autologous T cells was evaluated by using mAb to block the CD1w2 molecules expressed on the surface of cDCs. Incubation of cDCs with CD1w2-specific mAb followed by incubation with the same antibody conjugated to PE showed that the unlabeled CD1w2-specific mAb blocked binding of the PE-labeled anti-CD1w2 mAb by 54% (**Figure 11A**). In co-culture experiments conducted using cDCs pre-incubated with the CD1w2-specific mAb after stimulation with the lipid fractions and prior to the addition of autologous T cells, the expression of IFN-γ by CD4^+^ cells was considerably reduced as judged by reduction in MFC (**Figure 11B and C**). Taken together, these data suggest that CD1w2 molecules present on the cDCs play a role in activation of CD4^+^ T cells to produce IFN-γ *by B. pseudomallei* polar lipids.

## Discussion

While it has been long recognized that bacterial lipids are excellent diagnostic markers, we and others have demonstrated that bacterial polar lipids can also stimulate specific immune responses in the host [24], [31]. Here we demonstrated that polar lipids of *B. pseudomallei* (e.g. ornithine lipids and rhamnolipids) induce antibody production and that most polar lipids stimulate cellular immune responses. In this study, we fractionated polar lipids derived from the cell wall of *B. pseudomallei* strain K96243 and evaluated their capacity to stimulate antigen-specific responses by caprine CD4^+^ T cells given that goats are natural host for this bacteria. We obtained four major fractions (depicted in **Figure 1C** as F4, F5, F6, and F7) containing few or single polar lipids and analyses of lipids contained in each fraction by mass spectrometry revealed that fraction F4 contained ornithine lipids; fraction F5 contained ornithine lipids, rhamnolipids, and phospholipids (mainly phosphatidylethanolamine [PE]); fraction F6 contained ornithine lipids, PE lipids and lipid spot 19; whereas fraction F7 contained only lipid spot 19. While the chemical structures of rhamnolipids, ornithine lipids, and phospholipids of *B. pseudomallei* or closely related *Pseudomonas* ssp. were already determined by others [20], [21], [22], [32], [33], [34], [35], [36] the major focus of this study was to determine the involvement of polar lipids of *B. pseudomallei* in the induction of host immune responses. We tested the capacity of each one of these *B. pseudomallei* polar lipid fractions to stimulate immune responses in cell cultures derived from caprine peripheral blood white cells. We generated and used monocyte-derived caprine dendritic cells (cDCs), and showed that they displayed the classical DC morphology, and expressed CD14, MHC class II DR, and CD1w2 cell surface markers. While these markers are not exclusive for dendritic cells, together with the microscopic appearance of these cells, however, led us to conclude that these cells are truly dendritic cells. Thus, this is the first report to demonstrate that recombinant bovine cytokines (IL-4 and Flt3L/GM-CSF) stimulated caprine monocytes to differentiate to DCs. These data also demonstrated a preferential effect of *B. pseudomallei* polar lipids contained in fractions F4 – F7, on the surface expression of CD1w2 antigens than on the surface expression of MHC class II DR antigens by the cDC.

It is already known that CD1 molecules present mainly lipid antigens, whereas MHC class II DR molecules present peptides [25], [37], [38], [39], [40]. It is believed that activation of the protective responses mediated by polar lipids and the CD1 pathway has great advantages when compared to peptide presentation to T cells by the MHC. Thus, unlike the MHC pathway of antigen presentation, the CD1 pathway benefits from highly conserved CD1 antigen expression between species and individual subjects. Surprisingly, our data showed that interaction of each of the *B. pseudomallei* polar lipid fraction with the cDCs preferentially up-regulated the expression of CD1w2 molecules on the surface of the DCs but not the expression of MHC class II DR molecules. This capacity appears to allow the polar lipids contained in each of the lipid fractions to be recognized partly by CD1w2 restricted T cells and trigger expression of IFN-γ which is associated with protective immune responses. Altogether we believe these data have great potential within the vaccine development field.

Analyses of each fraction derived from *B. pseudomallei* demonstrated that fractions F4 to F7 contained only partial numbers of lipid moieties than the total lipids found in F1 and of the total polar lipids found in F2. Interestingly, the immunological responses elicited by lipids contained in fractions F4 to F7 also demonstrated a preferential capability to stimulate protective (IFN-γ) or immunosuppressive (IL-10) responses. While most studies on cellular immune responses induced by bacterial antigens have to eliminate endotoxins, preparation of lipid antigens through Folch wash eliminate the highly water soluble endotoxin and thus, we are certain that our data are not influenced by endotoxins. Fractions F6 and F7 induced much more IL-10 than IFN-γ, whereas F4 and F5 stimulated more IFN-γ than IL-10. Furthermore, the first (F1) and second (F2) extraction of *B. pseudomallei* lipids provided a more balanced immune response (IFN-γ and IL-10 cytokine expression), which is not a surprise since those are a combination of either all cell envelope lipids (F1) or contain all polar lipids including the polar lipids of the four fractions also tested (F2). The latter indicates that the effect on the cytokine responses stimulated in T cells by polar lipids is (a) lipid-specific and (b) depends on the amount present in the lipid cocktail. This is also supported by our data clearly demonstrating that ornithine lipids stimulate IFN-γ either alone as in fraction F4 or in combination with rhamnolipids and PE as in fraction F5. Ornithine lipids are found in a variety of bacterial species including various *Pseudomonas* species (*P. aeruginosa*, *P. stutzeri*, and *B. cepacia*]). Kawai and colleagues [30] reported previously that ornithine lipids derived from *Flavobacterium meningosepticum* are immune reactive and act as adjuvants to enhance immune responses when used with LPS of *B. cepacia*. The same authors also demonstrated that ornithine lipids protect mice from lethal endotoxemia [30], [40]. Thus, in our study the capacity of ornithine lipids derived from *B. pseudomallei* to stimulate IFN-γ responses is in line with those studies from Kawai and suggest that the ornithine lipids derived from *B. pseudomallei* may have potential use as adjuvants.

We also demonstrated that fraction F5 containing rhamnolipids and ornithine lipids did not stimulate IL-10. The lack of stimulation of IL-10 in cultures containing fraction F5 suggest that neither the ornithine lipids nor the rhamnolipids are good stimulators of IL-10 production when exposed to caprine cells. Rhamnolipids are known to be key cell surface molecule in various bacterial species including *Pseudomonas* ssp. and *Burkholderia* ssp. [32], [41]. Rhamnolipids are present in *B. pseudomallei* and they exhibit strong serological activities and induce cellular and functional changes in cell lines. Interestingly, the rhamnolipids from *B. pseudomallei* were found to be hemolytic for various erythrocyte species and to have cytotoxic effect for nonphagocytic and phagocytic cell lines [32], [33]. However, no data were provided yet until now that rhamnolipids from *B. pseudomallei* were capable of stimulating IFN-γ cytokine production by CD4^+^ T cells. Although we were unable to detect lipid A or deep rough LPS in our polar lipid fractions it has to be mentioned that rhamnolipids and perhaps ornithine lipids exhibit strong endotoxin-like properties and this might be the reason that both molecules induce IFN-γ. Several studies focused on the structural properties of glycolipids including lipid A, deep rough LPS, rhamnolipids and a glycoglycerolipid from *Mycoplasma fermentans* and how the chemical structure would contribute to the endotoxin-like properties of the lipids. While several of these lipids have acyloxyacyl moieties (rhamnolipids, hexaacyl Lipid A, heptaacyl Lipid A, haxaacyl deep rough LPS) and one might have hypothesized that this would be the key structural component leading to endotoxin-like properties, it was nicely shown that it is required for endotoxicity to have an amphiphilic lipid molecule with a nonlamellar cubic aggregate structure with a sufficiently high negative charge density in the saccharide-based backbone. This model would explain the endotoxicity of the glycoglycerolipid of *M. fermentans*, which does not have an acyloxyacyl structure but has the proposed nonlamellar cubic aggregate structure [42], [43], [44], [45].

Finally, our data suggest that lipid spot 19 found in fraction F6 and in fraction F7 is responsible for the IL-10 stimulation in caprine cells and that the inner membrane bacterial phospholipids (all are PE in our analyses) found in fractions F5 and F6 seemed not to contribute to the overall immune responses. However, PE may have a role in activation of B cells to produce antibody since in other studies it has been shown that purified PE induce antibody production [46]. In another study by Morgan and colleagues, murine peritoneal macrophages stimulated with PE induced TNF-α production [47] and may be implicated in activation of TLR. Lipid spot 19 seems to be an important immune suppressive polar lipid and might contribute to the overall immunosuppressive host responses during and after infection. While our data are a first indication for this hypothesis, further evaluation of the immune responses due to lipid spot 19 are needed to confirm this hypothesis. Furthermore, it has to be shown that this lipid spot is present in the lipid profiles of *B. pseudomallei* when grown intracellular and/or in vivo.

Future studies using goats that are either experimentally or naturally infected with *B. pseudomallei* will determine if the immunological properties for each polar lipid identified in these studies can be used as T cell biomarker of disease progression, as vaccine/adjuvant candidates or to develop cellular based immune detection strategies with lipids for diagnostic purposes. Moreover, further characterization of the polar lipids, their abundance, and association with immune response of the host will facilitate understanding of the pathogenesis of disease caused by pathogenic strains of *B. pseudomallei*. Because it is possible to chemically synthesize polar lipids, the use of polar lipids and their immunological properties represent a very promising venue to develop novel tools for the development of either vaccines or adjuvants, or could serve as biomarkers for melioidosis. In summary, our data demonstrated that among the polar lipids of *B. pseudomallei*, ornithine lipids and rhamnolipids are strong IFN-γ stimulators and thus both support the host immune response to control the disease, whereas the unknown polar lipid found in spot 19 of fraction F7 and F6 is on the opposite side as it strongly stimulates IL-10. No definitive role of the immune response for PE phospholipids could be determined.

The experiments in our study were designed as a proof-of-concept that the polar lipids of *B. pseudomallei* can induce strong cellular immune responses and that their presences or absences *in-vivo* needs to be evaluated. Although only two animals were used in this study, experiments were repeated several times to eliminate one-time effects. Our data provide convincing evidence that these polar lipids can contribute to pathogenicity and intracellular survival of *B. pseudomallei* during infection of at least goats if not humans.
